# Degradation of mitochondrial structure and deficiency of complex I were associated with the transgenic CMS of rice

**DOI:** 10.1186/s40659-020-00326-y

**Published:** 2021-02-22

**Authors:** Danfeng Tang, Fan Wei, Aziz Khan, Fazal Munsif, Ruiyang Zhou

**Affiliations:** 1 Guangxi Key Laboratory of Medicinal Resources Protection and Genetic Improvement, Guangxi Botanical Garden of Medicinal Plant, Nanning, 530023 China; 2 Guangxi Engineering Research Center of TCM Resource Intelligent Creation, Guangxi Botanical Garden of Medicinal Plants, Nanning, 530023 China; 3 College of Agriculture, Guangxi University, Nanning, 530004 China

**Keywords:** Rice, Transgenic CMS line, TEM, Mitochondrial biochemistry

## Abstract

**Background:**

Mitochondria play a significant role in plant cytoplasmic male sterility (CMS). In our previous study, mitochondrial complex I genes, *nad4*, *nad5*, and *nad7* showed polymorphisms between the transgenic CMS line M2BS and its wild type M2B. The sterility mechanism of the M2BS at cytological, physiological, biochemical, and molecular level is not clear.

**Results:**

Cytological observation showed that the anthers were light yellow, fissured, invalid in KI-I_2_, and full of irregularly typical abortion pollen grains in M2BS. Transmission electron microscopic (TEM) observation revealed no nucleus and degraded mitochondria with obscure cristae in anther cells of M2BS. The results of staining for H_2_O_2_ presented a large number of electron dense precipitates (edp) in intercellular space of anther cells of M2BS at anthesis. Moreover, the anther respiration rate and complex I activity of M2BS were significantly lower than those of wild type M2B during pollen development. Furthermore, RNA editing results showed only *nad7* presented partially edited at 534th nucleotides. The expression of *nad5* and *nad7* revealed significant differences between M2B and M2BS.

**Conclusions:**

Our data demonstrated that mitochondrial structural degradation and complex I deficiency might be associated with transgenic CMS of rice.

## Background

Cytoplasmic male sterility (CMS) was maternally inherited in plants and in most cases originated from mitochondrial DNA rearrangements which resulted in plants inability to produce functional pollen [1–5]. The CMS trait was observed in more than 200 flowering plant species [6, 7]. CMS produced hybrids using “three-line” system can show heterosis, which exhibits the improved performance of hybrid progeny in comparison with the parental lines [8]. For example, the yield had increased up to 20% in hybrids of rice by using CMS lines [9, 10].

Generally CMS was associated with mitochondria, which was an essential organelle for cellular energy production [10, 11]. Since pollen development required a large amount of energy supply, disturbances or disorder in mitochondrial functions could have important effects on male fertility [5, 12]. The mitochondrial respiratory chain consisted of four complexes: NADH dehydrogenase complex (Complex I), succinate dehydrogenase complex (Complex II), cytochrome c reductase complex (Complex III), and cytochrome c oxidase complex (Complex IV). The F_0_F_1_-ATP synthase was Complex V. In mitochondria, energy was generated by the production of proton gradient via electron transport, which catalyzed electron transfer from NADH or FADH2 to molecular oxygen and lead to ATP generation [13]. Any disturbance of this electron transport process could impair the energy production [10].

Mitochondrial complex I was an important pathway for the oxidation of NADH in bacteria, higher plants and animals, and had to do with plant CMS. In plants, CMS phenotype has been described involving *nad9* and *nad7*. For instance, the rice CMS line RT98A derived from *Oryza rufipogon*, the *orf113* displayed completely identical sequences to *nad9* in the region − 151 bp to + 11 bp, whereas the rest consisted of unknown sequences [14]. In *Nicotiana sylvestris* CMS I and CMS II, deletion of exons III and IV of *nad7* in CMS I and the complete disappearance of *nad7* in CMS II resulted in CMS [15].

In our previous study, mitochondrial complex I genes, *nad4*, *nad5*, and *nad7* showed polymorphisms between M2B and M2BS [16]. In the present study, in order to further understand the relationship between these genes and CMS characteristics in M2BS, the anther cytological observation were performed, the mitochondrial complex I activity and respiration rate were determined, and the RNA editing and the relative expression of these genes were also analyzed between the two lines.

## Materials and methods

### Plant materials

M2BS was a transgenic CMS line induced by the partial-length *HcPDIL5-2a* transformation in rice [16] using M2B as a transgenic receptor material. M2B was an *indica* rice variety and was the maintainer line of M2BS. After successively backcrossing, all the plants were planted in the field with normal management.

### Optical microscopic observation

Anthers morphologic observation was performed by camera (*Cannon*, Japan). Anthers were harvested from upon, middle and bottom spike of three plants and were stained with 1% I_2_-KI to observe pollen fertility at anthesis.

### Scanning electron microscope (SEM)

Anthers were collected at anthesis and fixed for 24 h in Carnoy's Fluid, then dehydrated by a standard series of ethanol washes: 70%, 80%, 90%, and 100% each for 5 min, immediately dried for several minutes, coated with gold by a gold sputter, and was analyzed using Phenom LE electron microscope.

### Transmission electron microscope (TEM)

The anthers on the top portion of spikes were used and placed in a bottle contained 2.5% glutaraldehyde buffer solution at anthesis. Air was pumped out of centrifuge tubes in order to soak anthers fully in buffer solution. Anthers were fixed, dehydrated, embedded and examined according to the reference [17].

### Cytochemical detection of H_2_O_2_

H_2_O_2_ was visualized at the subcellular level using CeCl_3_ for localization [18]. Electron-dense CeCl_3_ deposits are formed in the presence of H_2_O_2_ and are visible by transmission electron microscopy. Anthers were excised from CMS line and maintainer line at anthesis and incubated in freshly prepared 5 mM CeCl_3_ in 50 mM MOPS at pH 7.2 for 1 h. The experimental method was referred to [19].

### Determination of mitochondrial complex I activity and respiration rate

Spike samples from booting stage and anthesis were collected and stored at − 80 ℃ for determination of biochemical attributes. The activity of mitochondrial complex I was determined using mitochondrial complex I Kit (COMIN, Suzhou, China). The experimental operation was completed according to the kit instructions of manufacturer. Calculated by the fresh weight of sample, each gram organization consumes 1 nmol NADH per minute, defined as an enzyme activity unit. The formula for measuring using 96 orifice plates was as follows: Mitochondrial complex I activity (U/g fresh weight) = [ΔA × Vt ÷ (ε × d) × 10^9^] ÷ (W × Vs ÷ Vts) ÷ T = 730 × ΔA ÷ W. Vt: total volume of reaction system, 2.25 × 10^–4^ L; ε: NADH molar extinction coefficient, 6.22 × 10^3^ L/mol/cm; d: diameter of 96 orifice plates, 0.5 cm; Vs: sample volume added, 0.01 mL; Vts: the added volume of extract solution, 0.202 mL; T: reaction time, 2 min; W: sample weight, g. The respiration rate of anthers was determined by the method of Balkos et al. [20].

### RT-PCR amplifications

Total RNA samples were isolated from young panicles with CTAB as described by Liu and He [21]. First-strand complementary DNAs (cDNAs) were synthesized from 1 μg RNA using PrimeScript RT reagent Kit with gDNA Eraser (TaKaRa Bio-medicals, Dalian, China). Elimination of DNA was checked by PCR for N4 primers, which amplified a 463 bp fragment for cDNA and a 1717 bp fragment (including a 1254 bp intron) for gDNA (Additional file 1: Fig. S1). The PCR amplifications were carried out using a thermal cycler (Bio-Rad, Hercules, CA, USA) in a total volume of 25 μL reaction mixture, which included 12.5 μL 2 × Mix (containing high-fidelity DNA polymerase) (Takara, Dalian, China), 6.5 μL ddH_2_O, 2 μL cDNA, 2 μL each primer (10 μmol/L each). The primer sequences were listed in Additional file 2: Table S1. The PCR conditions were as follows: an initial denaturation step at 94 °C for 3 min, followed by 34 cycles of denaturation at 94 °C for 30 s, annealing at 55 °C for 30 s, and extension at 72 °C for 1 min, followed by a final extension at 72 °C for 5 min.

### Sequencing and calculation of editing frequency

The PCR products were eluted, purified, cloned and last sequenced by Beijing Genomics Institute (BGI). Editing efficiency was determined by totally sequencing at least 12 cDNA clones from two separate experiments. Editing frequency was calculated by the ratio of clones containing this editing to the total clones sequenced [22]. The nucleotide sequences were analyzed through DNA Man soft program and submitted to ORF finder and NCBI online service. PCR primers used in this study are listed in Additional file 2: Table S1.

### Quantitative reverse transcription PCR (qRT-PCR) analysis

qRT-PCR was performed with qTOWER2.2 sequence detection system (Jena, Germany) using SYBR® Premix Ex Taq™ (Takara, Dalian, China). A housekeeping gene glyceraldehyde-3-phosphate dehydrogenase (*GAPDH*) of rice served as the internal reference. The primer pairs RT-ND5-F/R, RT-nad4-F/R, RT-nad7-F/R, RT-GAPDH-F/R were used for qRT-PCR analysis at anthesis (Additional file 2: Table S1). All reactions were conducted in triplicate for each sample. The relative expression spikes of each transcript were estimated by the 2^−△△Ct^ method [23].

## Results

### Morphological observation of anther

**Fig. 1:**
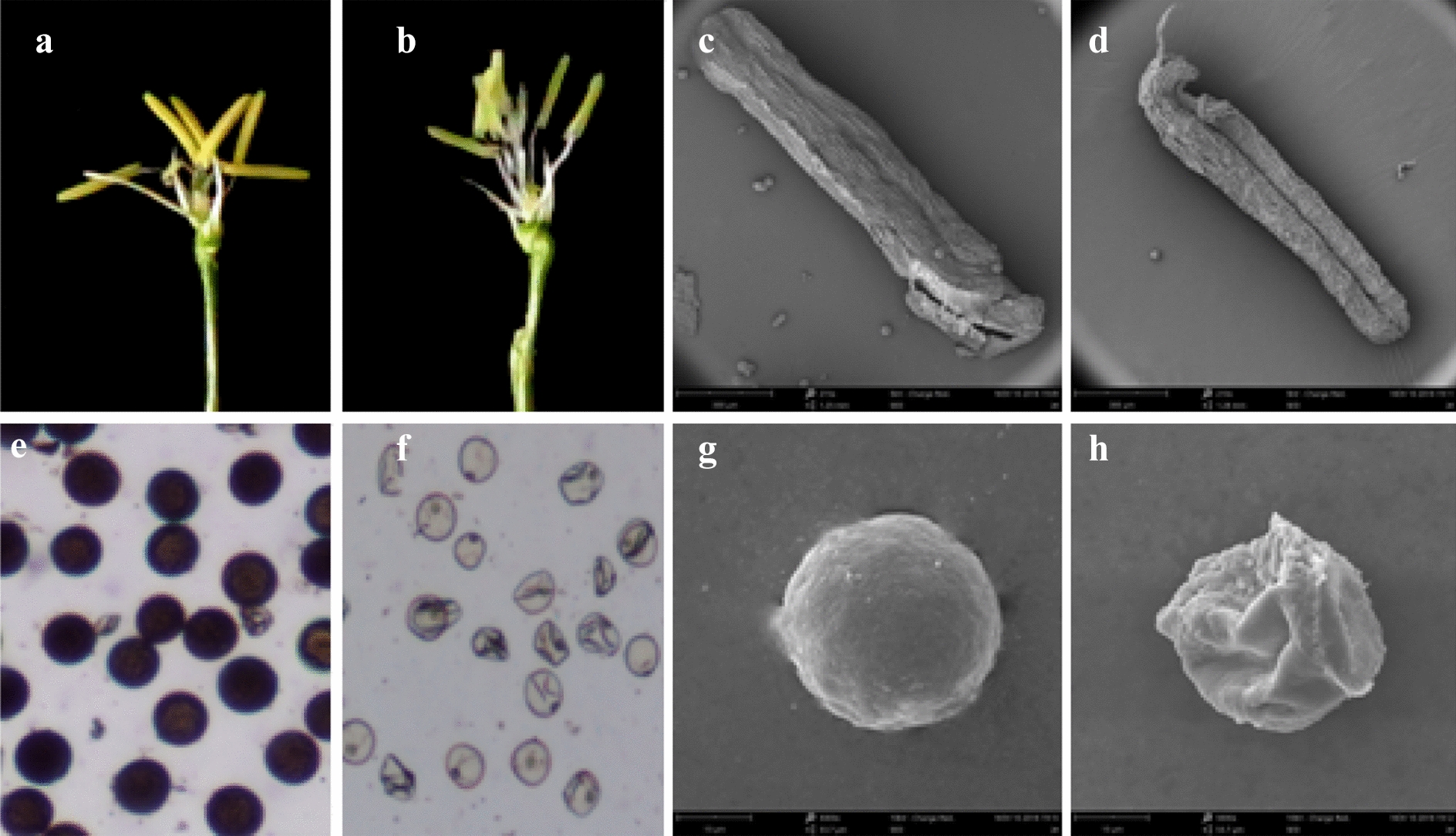
Morphological characteristics of anthers. **a**, **c** represented the anther of M2B; **b**, **d** represented the anther of M2BS; **e**, **g** represented the pollen of M2B; **f**, **h** represented the pollen of M2BS. 10 ×

The maintainer line (M2B) anther was yellow (Fig. 1a), while the anther of the CMS line (M2BS) were light yellow (Fig. 1b). The pollen grains of M2B could be stained by KI-I_2_ and were dark grey (Fig. 1e), whereas the sterile pollen grains were invalid in KI-I_2_ (Fig. 1f). SEM results showed sturdy anther and spherical pollen grain in M2B (Fig. 1c, g). However, M2BS presented fissured anther and irregularly typical abortion pollen grain (Fig. 1d, h).

### Transmission electron microscopy analysis

**Fig. 2:**
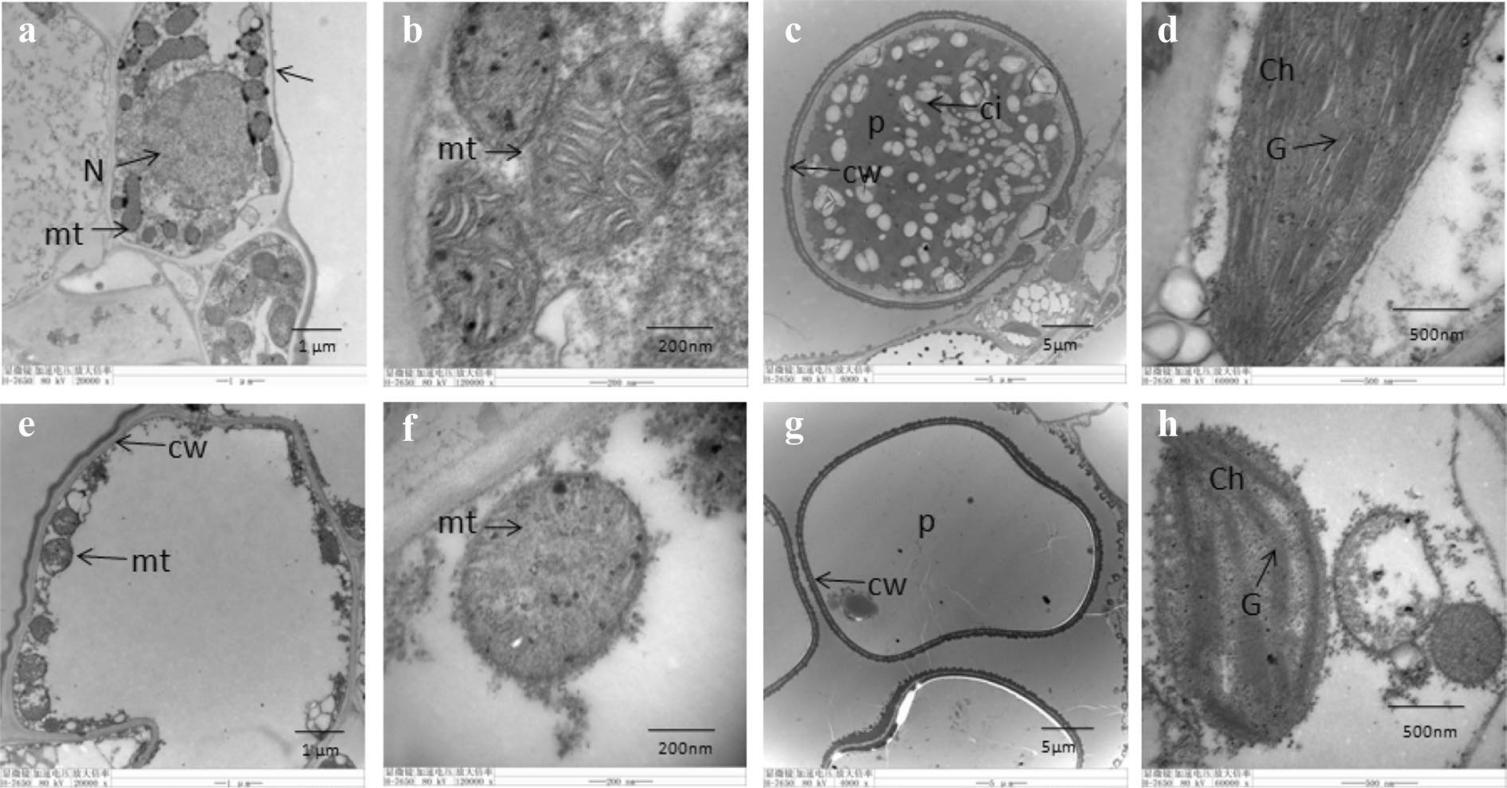
Transmission electron micrographs of anther mitochondria and chloroplast ultrastructure at anthesis. **a**-**d** represented maintainer line M2B; **e**–**h** represented CMS line M2BS; **a**, **e** Nucleus observation; **b**, **f** Mitochondria observation; **c**, **g** Mature pollen grains observation. **d**, **h** represented anther chloroplast ultrastructure of M2B and M2BS, respectively. N: Nucleus; mt: Mitochondria; Ch: Chloroplast; P: Pollen grains; cw: cell wall. G: Granal thylakoids

Studies reported that mitochondrial ultrastructure was involved in plant CMS [24]. In this study, TEM was performed to study the mitochondrial ultrastructure changes in two lines. The results showed intact nucleus in M2B anther cells, whereas the nucleus of M2BS exhibited degradation and disappearance (Fig. 2a, e). The anther mitochondria of M2B revealed intact mitochondrial structure and clear ridge. In contrast, the mitochondria degraded to some extent, and the cristae of mitochondria were obscure and disintegrated partly in M2BS (Fig. 2b, f). The mature pollen grains of M2B showed circular shape, while M2BS presented non-circular pollen grains. In M2B pollen grains, we observed the cellular inclusions, while nothing was observed in that of M2BS (Fig. 2c, g). Moreover, the intact chloroplast structure, dense and clear thylakoids were observed in anther cells of M2B (Fig. 2d). However, anther chloroplast structure of M2BS was fuzzy and degraded partly, and the thylakoids dissolved (Fig. 2h).

### Subcellular localization of H_2_O_2_ in anther

**Fig. 3:**
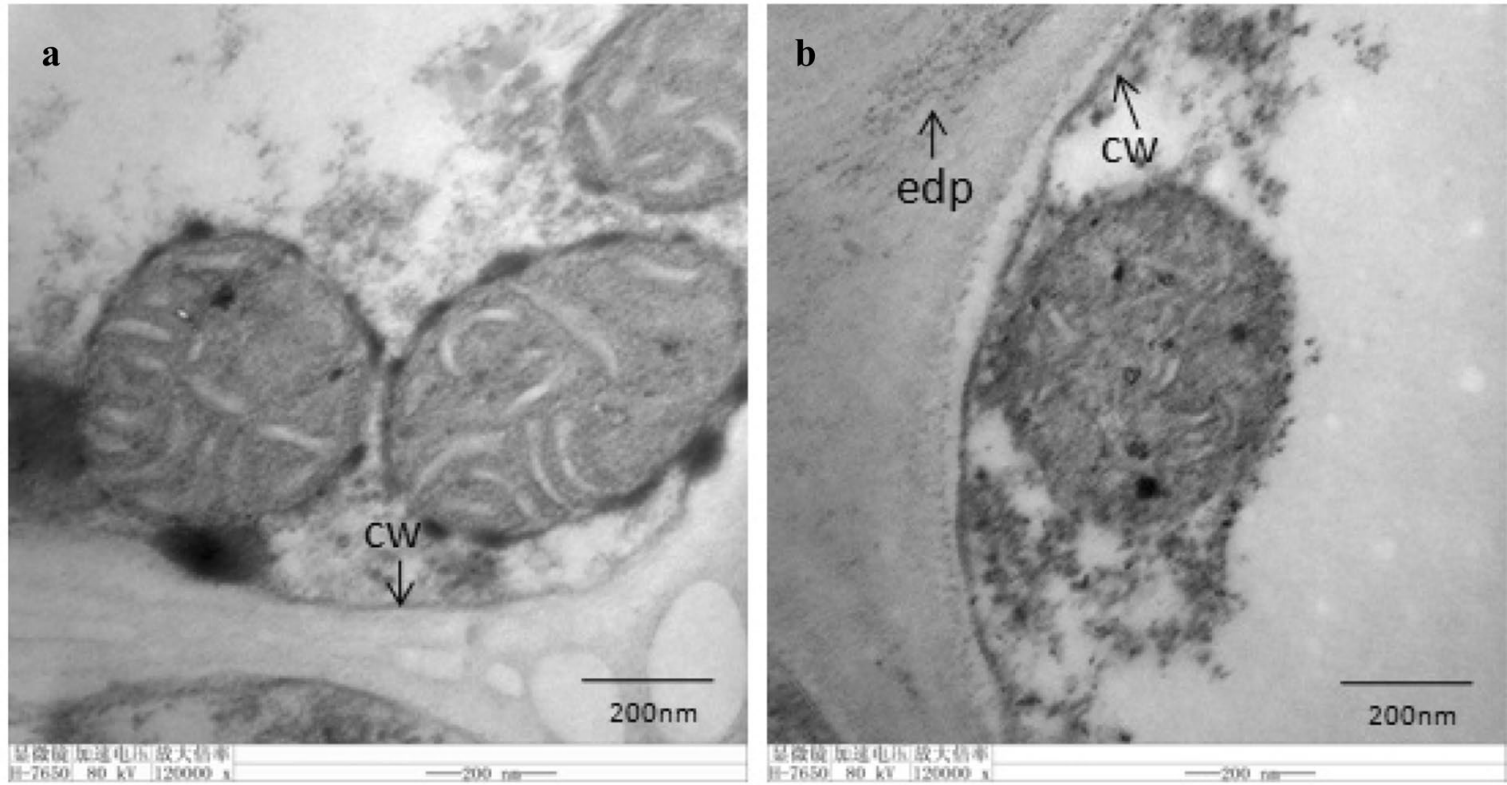
The sites of electron-dense precipitates of anther at anthesis. **a**, **b** represented M2B and M2BS, respectively. edp: electron-dense precipitates; cw: cell wall

There were a lot of evidences that plant CMS was accompanied by the production of large amounts of reactive oxygen species (ROS) [12], and it could be observed that the combination of H_2_O_2_ and CeCl_3_ could form the electron-dense precipitates at transmission electron microscope (TEM). In the present study, the sites of electron-dense precipitates of CMS line and its maintainer line were observed in anther cell at anthesis. The results showed that a large number of electron dense precipitates appeared in intercellular space of anther cells of M2BS at anthesis (Fig. 3b), whereas no obvious electron dense precipitates were observed in those of M2B (Fig. 3a).

### Changes of respiration rate and mitochondrial complex I activity

**Fig. 4:**
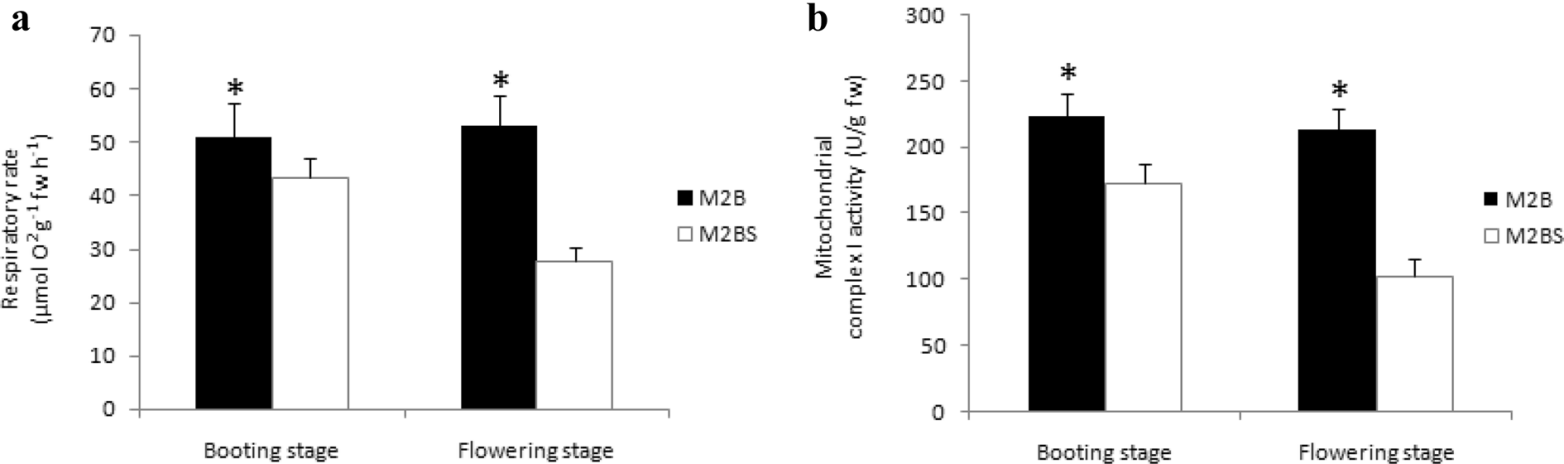
Respiration rate and mitochondrial complex I activity during different pollen development stages

Mitochondria is the center for plant energy biology and plays a vital role in the respiration and metabolism of plants. In the present study, the spike respiration rate of two lines were determined both at booting stage and anthesis. The results indicated that the respiration rate of M2B presented no obvious changes at different stages of anther development. However, significant decrease was detected in M2BS at anthesis. Compared with M2B, the respiration rate at booting stage and flowering stage decreased by 14.9% and 47.8% in M2BS, respectively (Fig. 4a).

In order to understand the complex I probably associated with CMS characteristics, the activity of complex I was investigated to determine how a partially mitochondrial and partially nuclear encoded enzyme reacted to this cytoplasmically determined phenomenon of CMS. The complex I showed a significantly reduced activity in M2BS, compared to M2B during anther development process. The complex I activity of M2BS dramatically decreased by 22.8% and 51.9%, respectively at booting stage and flowering stage (Fig. 4b).

### RNA editing and expression of *nad4, nad5*, and *nad7*

**Fig. 5:**
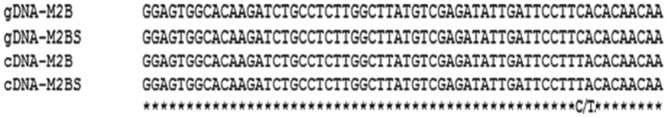
Editing site of *nad7*

RNA editing result showed only *nad7* presented partially edited among these three genes. The CDS region of *nad7* was verified using primers of nad7cds-F and nad7cds-R, a specific product approximately 1185 bp was amplified from cDNA of M2B and M2BS, respectively (Additional file 3: Fig. S2). The comparisons of cDNA and genomic sequence data revealed only one distinct editing site in the *nad7* CDS region of M2B and M2BS (Fig. 5). This common editing site located at 534th nucleotides with the initiation codon of *nad7*. The editing efficiency was shown in Table 1, and the results showed that the editing frequencies were higher in M2BS than in M2B. The nucleotide substitutions were C-U transitions for this editing site in *nad7* transcripts, where was a silence modification.

**Table 1:** Editing frequencies of *nad7* transcripts in M2B and M2BS

Materials	Numbers of Clones(534th)		Editing frequency (%)
	C	T	
M2B	6	6	50.0%
M2BS	5	7	58.3%

The site numbers are determined by the nucleotide positions with the initiation codon of *nad7,* where editing occurs. The 534th editing site is C-U conversion

**Fig. 6:**
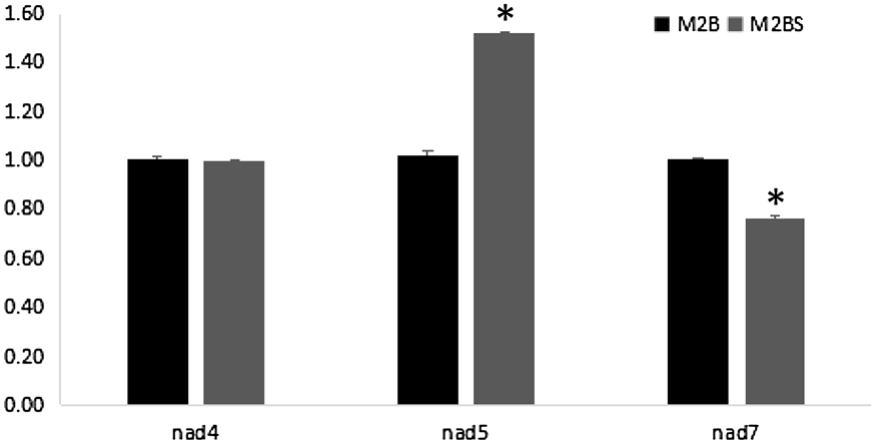
The relative expression of *nad4, nad5,* and *nad7*

In order to further study the transcriptional expression of *nad4, nad5*, and *nad7* at anthesis, we performed qPCR for M2B and M2BS. The results showed that The expression of *nad5* and *nad7* revealed significant differences between two lines (Fig. 6).

## Discussion

Mitochondria is an essential organelle in cell not only because it supplies over 90% energy for cells but also because its dysfunction is associated with disease [25]. As the main organelle of energy metabolism, the structure and quantity of mitochondria might be related to male sterility [24]. Changes in mitochondrial structure lead to abnormal electron transfer chains or subunit complexes of ATP enzymes involved in energy metabolism in mitochondria, resulting in the absence of sufficient energy supply for microspore development and pollen abortion [26]. In the present study, the disappearance of cell nucleus and the abnormal mitochondrial structure were observed in anthers of CMS line (Fig. 2). It was in accord with previous studies [27–29]. These findings may support the opinion that abnormal mitochondrial structure may be the cellular morphology characteristics of male sterility. In addition, a large number of electron dense precipitates appeared in intercellular space of anther cells of M2BS (Fig. 3b). It was indicated that the H_2_O_2_ burst and the production of these ROS at anthesis would also have influences on pollen development and growth. These findings were consistent with the results observed in *Brassica napus* [30].

Mitochondria was the site of aerobic respiration. Respiration rate was an important physiological attribute for identifying plants respiration intensity and energy metabolism [31]. Previous studies showed lower respiration rate in male sterile anthers compared with fertile ones, indicating some defects in some steps of respiration in male sterile anthers [32–35]. In this study, the respiration rate of the CMS line was lower than that of the maintainer line (Fig. 4a), which was in agreement with the reports mentioned above.

Mitochondrial complex I was one of the largest macromolecular complexes [36, 37] which played an important role in the cellular energy production. Complex I, a functional enzyme, generated reactive oxygen species (ROS), which could be detrimental, but was also of importance for cell signaling [38]. Defects in this enzyme lead to a severe disturbance of energy metabolism and often lead to severe inherited metabolic disorders [39]. Ducos et al. [40] found that the NAD9 subunit had a C-terminal extension while COX2 subunit had a truncated C-terminus in two mutations of CMS wild beet. Further, they reported that the complex I activity was unchanged in leaves, but the complex IV activity was reduced by 50%. In the present investigation, the different result was obtained and the lower complex I activity was detected in M2BS (Fig. 4b).

CMS and RNA editing were two important phenomena involving in plant mitochondria. It was generally agreed that CMS was caused by altered gene expression due to defective or inadequate RNA editing [22, 41–43]. Kim et al. found defects at seven specific editing sites in five mitochondrial genes (*cox2*, *cox3*, *nad2*, *nad4* and *ccmc*) of an ogr1 rice mutant, which were in connection with pollen grain abnormalities [44]. Recently, editing was absent in *nad4* at position 1033 of mutant plants, which carried a cytidine residue at this position, where wild type plants instead carried a uridine residue [45]. Compared to Weibenberger et al. [45], in the present study, the nucleotide substitutions were all C-U transitions for the editing site in *nad7* transcripts, where was a silence modification site (Fig. 5) and the expression of *nad5* and *nad7* revealed significant differences between two lines (Fig. 6). Therefore, it was inferred that the mutation, RNA editing, and expression of the complex I genes may be associated with the CMS. In our previous study, the results showed chloroplast differences in leaves of two lines, such as photosynthetic parameters, chloroplasts ultrastructure, soluble sugar and starch content, sugar and starch metabolism genes expression, and photosynthetic related genes [46]. As is known to all, chloroplast is an important site for the photosynthesis and mitochondria is the place of respiration. Photosynthesis uses light energy and water to convert atmospheric carbon into carbon-rich compounds such as carbohydrates. Respiration oxidizes these compounds, releasing useable energy and forming carbon intermediates needed for biosynthesis. On a whole plant basis, up to 70% of the fixed during photosynthesis can be released back into the atmosphere by mitochondrial respiration [47]. Therefore, a further understanding of both photosynthesis (chloroplast) and respiration (mitochondria), and the interplay between them, is necessary.

## Conclusion

Morphological and cytological observations showed significant differences between M2BS and M2B. The anther respiration rate and complex I activity of M2BS were significantly lower than those of M2B during pollen development. The expression of *nad5* and *nad7* revealed significant expression differences between two lines. Overall, the mitochondrial structural degradation and complex I deficiency might be associated with the transgenic CMS of rice.

## Supplementary Information

**Additional file 1.** Amplification of gDNA and cDNA with prime N4-intron.

**Additional file 2.** Primers used in the present study.

**Additional file 3.** PCR amplification bands of *nad7* CDS region in M2B and M2BS.

## Data Availability

Not applicable.

## Abbreviations

CMS: Cytoplasmic male sterility
TEM: Transmission electron microscopic
SEM: Scan electron microscopic
qRT: Quantitative reverse transcribed PCR
