# Supplementary material for: Degradation of mitochondrial structure and deficiency of complex I were associated with the transgenic CMS of rice
Source: Biol Res. 2021 Feb 22;54:6. doi: 10.1186/s40659-020-00326-y (PMC7898427; doi:10.1186/s40659-020-00326-y)
Supplement: Supplementary file 1 — Additional file 1. Amplification of gDNA and cDNA with prime N4-intron. [file 40659_2020_326_MOESM1_ESM.docx]

| Table S1 Description of primers used in the study: F, forward primer; R, reverse primer. | | | |
| --- | --- | --- | --- |
| Primes | Base sequence 5’ to 3’ | Tm(℃) | Description |
| nad7cds-F | ATGACGACTAGGAACGGGCAAATCA | 55 | PCR primer for amplification the full-length of *nad7* gene |
| nad7cds-R | CTATCTATCTACCTCTCCAAACACAATATCT | 55 |  |
| N4-intron-F | TGCGTTTCTCTTATTACTTTTTTGT | 52 | PCR primer for detecting intron |
| N4-intron-R | TTCCTGTTTGGAGAAGAATCAACA | 52 |  |
| RT-ND5-F | ACTTTCTCCAATTATTCCTGGG |  | Primers for qPCR |
| RT-ND5-R | TTATAGCTGCTTTATCCGCCTG |  |  |
| RT-nad4-F | TTCTTCTCCAAACAGGAACCACC |  | Primers for qPCR |
| RT-nad4-R | AAGAGGCGAAAAAAGCAATCCAT |  |  |
| RT-nad7-F | ATTCTTCAGCCGTAGAGAGACTTT |  | Primers for qPCR |
| RT-nad7-R | CCACATCCATAGCATGAGTAGTTG |  |  |
| RT-GAPDH-F | TTTTGGGCTGCAGGGATGTG |  | Primers for qPCR |
| RT-GAPDH-R | TCCTCGAGCCGGTGCAGAGT |  |  |

| Table S2. Editing frequencies of *nad7* transcripts in M2B and M2BS. | | | | | |
| --- | --- | --- | --- | --- | --- |
| Materials | Numbers of Clones(534^th^) | | |  | Editing frequency(%) |
|  | C |  | T |  |  |
| M2B | 6 |  | 6 |  | 50.0% |
| M2BS | 5 |  | 7 |  | 58.3% |
| M2A | 3 |  | 9 |  | 75.0% |

The site numbers are determined by the nucleotide positions with the initiation codon of *nad7* where editing occurs. The 534^th^ editing site is C-U conversion.
