# Supplementary material for: Degradation of mitochondrial structure and deficiency of complex I were associated with the transgenic CMS of rice
Source: Biol Res. 2021 Feb 22;54:6. doi: 10.1186/s40659-020-00326-y (PMC7898427; doi:10.1186/s40659-020-00326-y)
Supplement: Supplementary file 2 — Additional file 2. Primers used in the present study. [file 40659_2020_326_MOESM2_ESM.docx]

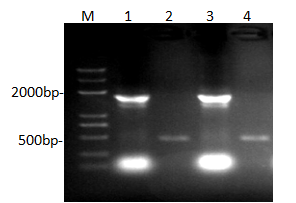


Additional files 2, Intron detection of gDNA and cDNA with prime N4-intron. Lane1, 3 was a 1717 bp products with gDNA as template in M2B and M2BS. Lane2, 4 was a 463 bp product with responding cDNA, M: BM 5000 DNA marker.
