# Supplementary material for: Degradation of mitochondrial structure and deficiency of complex I were associated with the transgenic CMS of rice
Source: Biol Res. 2021 Feb 22;54:6. doi: 10.1186/s40659-020-00326-y (PMC7898427; doi:10.1186/s40659-020-00326-y)
Supplement: Supplementary file 3 — Additional file 3. PCR amplification bands of nad7 CDS region in M2B and M2BS. [file 40659_2020_326_MOESM3_ESM.docx]

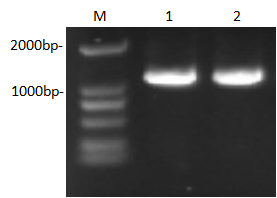


Additional files 3, The CDS region of *nad7* of M2B and M2BS. Lane1,2 represented M2B and M2BS, respectively; M: BM2000 DNA Marker.
